# PD156 Scanning The Right Horizons: Does Singapore’s Horizon Scanning Identify And Assess The Relevant Technologies?

**DOI:** 10.1017/S026646232400388X

**Published:** 2025-01-07

**Authors:** Jaryl Ng, Hong Ju, Zhen Long Ng, Swee Sung Soon, Kwong Ng

## Abstract

**Introduction:**

In Singapore, a horizon scanning (HS) system was established in 2020 by the Agency for Care Effectiveness (ACE) to provide early alerts of new and emerging medical technologies (medtechs) for service planning and to warn against the diffusion of low value technologies. This study compared the medtechs identified and assessed by ACE with health technology trends identified by our reference agency.

**Methods:**

Medtechs identified and assessed by ACE between 2020 and 2023 were analyzed to examine their distribution. Most of the identified medtechs were classified as digital health technologies, precision medicine, robotics, or implants. The prioritized technologies with a completed in-depth assessment were compared with the top 10 health technology trends identified in 2022 by our reference health technology assessment (HTA) agency, the Canadian Agency for Drugs and Technologies in Health (CADTH). Additionally, feedback from key stakeholders such as clinicians and policymakers on the HS reports was summarized to understand the relevance and value of the HS reports.

**Results:**

From 2020 to 2023, there were 1,703 medtechs identified from various databases and manufacturer submissions. Digital health technologies were the largest proportion of technologies identified during this period, increasing from 26 percent in 2020 to 31 percent in 2023. Of the 20 evaluated technologies, 70 percent belonged to the top trending medtech fields identified by CADTH. These included artificial intelligence for diagnostics (n=5), point-of-care testing (n=4), companion diagnostics (n=2), wearables (n=2), and remote monitoring (n=1). Initial stakeholder feedback was positive, citing HS reports as being relevant for clinical practice. Some HS reports were also referenced by other policymakers to support regulatory decisions.

**Conclusions:**

In summary, similarities were observed between medtechs identified and assessed by the ACE HS system and the top trending medtech fields identified by CADTH. Additionally, digital health technologies were the largest proportion of technologies identified by the ACE HS system in 2023. This was substantiated by feedback from our key stakeholders, indicating the relevance and value of the ACE HS work.

